# Left Ventricular Thrombus in Ischemic Heart Failure: Machine-learning–based Prediction of Six-month Persistence and One-year Outcomes

**DOI:** 10.1007/s12265-026-10804-5

**Published:** 2026-06-22

**Authors:** Yunus Emre Yavuz, Yakup Alsancak, Sefa Tatar, Hayri İncekara, Büşra Özyeşi̇l, Hakan Akilli, Abdullah İçli̇

**Affiliations:** 1 Department of Cardiology, Meram Faculty of Medicine, Necmettin Erbakan University, Konya, Turkey; 2 Republic of Türkiye Ministry of National Education, Konya, Turkey

**Keywords:** Left ventricular thrombus, Machine learning, Ischemic heart failure, Echocardiography

## Abstract

Left ventricular thrombus (LVT) in ischemic heart failure carries embolic risk; tools to anticipate persistence are limited. We studied 190 consecutive patients with imaging-confirmed LVT managed with guideline-concordant anticoagulation and serial echocardiography. The primary outcome was 6-month non-regression; 1-year MACE was secondary. We combined classical statistics with explainable machine learning. CatBoost yielded the best discrimination for non-regression (CV-AUC 0.76; test accuracy 0.79). SHAP highlighted left atrial diameter, pulmonary artery pressure, platelet count, and LV end-diastolic diameter as leading predictors. For 1-year outcomes, thrombus size and CHA2DS2-VA were independently associated with MACE (logistic AUC 0.71), whereas “regression vs persistence” alone was not. Baseline remodeling and coagulability markers, captured by an interpretable ML model, stratify early risk of LVT persistence and complement clinical decision-making for imaging follow-up and anticoagulation intensity.

**Graphical Abstract:**

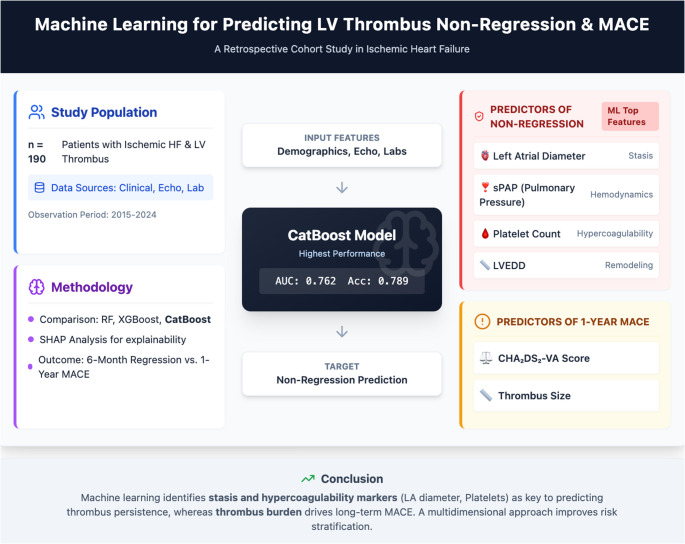

## Introduction

Left ventricular (LV) thrombus is a clinically important complication in ischemic heart disease/heart failure, associated with a markedly increased risk of systemic embolism and adverse cardiovascular outcomes [1]. Echocardiography remains the first-line imaging modality for detection and follow-up of LV thrombus in routine care, whereas contrast-enhanced echocardiography and cardiac magnetic resonance can provide diagnostic certainty and characterize thrombus burden and morphology [2]. Typical management consists of systemic anticoagulation for several months with interval imaging until resolution is documented [3, 4].

Machine learning (ML) is a powerful predictive tool for identifying intracardiac thrombosis and high-risk patients. In the literature, ML has been shown to be effective for predicting the severity of coronary artery disease/CAD-RADS, forecasting in-hospital outcomes in STEMI, estimating mortality risk after Transcatheter Aortic Valve Implantation, and predicting residual thrombotic risk despite anticoagulation in atrial fibrillation [5–8]. Collectively, these studies indicate that by simultaneously capturing the contributions of multiple clinical/demographic variables, ML strengthens risk stratification, supports personalized management, and facilitates earlier intervention with potential improvements in patient outcomes.

Among patients with ischemic heart failure and confirmed LV thrombus, a non-negligible subset exhibits incomplete regression or persistent thrombus despite guideline-concordant therapy, thereby sustaining concern for embolic events and prolonged anticoagulation. Robust, pragmatic tools to anticipate non-regression at diagnosis or soon thereafter remain limited [9]. Accordingly, in a cohort of ischemic heart failure with confirmed LV thrombus, we compared baseline demographic, clinical, laboratory, and echocardiographic characteristics according to six-month thrombus regression under effective anticoagulation, and aimed to develop a machine-learning model to predict the subgroup with non-regression.

## Methods

### Study Design and Population

This was a single-center, retrospective, observational cohort study. Ethical approval was received for the study from the ethics committee of Necmettin Erbakan University Faculty of Medicine with reference number 2025/5962. Patients admitted to our clinic between 2015 and 2024 were examined. Inclusion criteria: left ventricular (LV) thrombus confirmed by echocardiography on the basis of ischemic heart failure and follow-up under effective anticoagulation after diagnosis. All patients received warfarin; INR was monitored and maintained within the therapeutic range per institutional protocol. Exclusion criteria; missing principal variables that disrupted data integrity and facts without outcome information. Follow-up was based on the “baseline” evaluation at the time of diagnosis, 6-month follow-up imaging, and 1-year clinical follow-up. Regression was defined as the documentation of complete disappearance of the left ventricular thrombus in any of the serial echocardiography. In the analyses, all patients with regression detected within 6 months were combined in a single group (Group 1); Patients with persistent thrombus at the end of 6 months or no documented regression within 6 months were classified as Group 2.

### Echocardiography Measurements and Laboratory

Transthoracic echocardiography was used for diagnosis and serial follow-up. Thrombus size was determined by the field method (planimetry) in routine clinical practice. Indicators of left ventricular function and remodeling (e.g., LVEF, LVEDD/LVESD), left atrium (LA) diameter, and pulmonary artery pressure (PAB) were reported according to standard guideline measurement techniques. Laboratory parameters (e.g., platelet count) were recorded to match the time period of diagnosis.

### Outcomes

The primary comparison was based on whether there was thrombus regression (Group 1) or no (Group 2) at 6 months despite effective anticoagulation. The primary predictive model targeted the risk of non-regression at 6 months. The secondary clinical outcome was 1-year MACE (major adverse cardiovascular events) as defined in the dataset, and independent association analysis was also conducted for this outcome.

### Statistical Analysis

Normally distributed variables were determined in Mean ± SD format with independent sample t test; those that are not normally distributed were presented in median (min–max) format with Mann–Whitney U. Categorical variables were compared with Pearson chi-square (Fisher exact test if necessary) in the form of n (%). In the prediction models, logistic regression was applied for the 6-month regression and 1-year MACE. The coefficients were given by odds ratio (OR) and 95% confidence interval (CI). Distinctiveness was assessed by ROC-AUC. Marginal prediction curves (g-computation) and 2-dimensional risk heat maps were produced for clinical visualization. Considering the number of events (events-per-variable), the models were kept partially. Multicollinearity among candidate echocardiographic predictors was evaluated using correlation matrices and variance inflation factors (VIF), with VIF > 5 considered suggestive of problematic collinearity.

### Machine Learning Sub-analysis

The aim of the sub-analysis was to predict thrombus non-regression (Group 2) with baseline variables at 6 months. The result variable was coded in pairs (0 = regression, 1 = non-regression). All available demographic, clinical, laboratory, and echocardiographic baseline variables were included as candidate predictors. The process was conducted as follows: (i) the missing values were completed with median assignment; (ii) continuous variables were scaled, categoricals were label coded; (iii) the data was divided into 80%/20% stratified training-testing; (iv) model selection and hyperparameter optimization were performed with 5-fold stratified cross-validation in the training set; (v) Stratified sampling/balanced weighting was applied for class imbalance. Algorithms compared: Random Forest, XGBoost, CatBoost (binary logistic objective function). The primary performance criteria were ROC-AUC, and the secondary criteria were accuracy, recall, specificity, precision and F1. Permutation-significance and SHAP were used for feature significance; In addition, direction/severity was examined with partial dependency curves when necessary.

## Results

### Study Population

A total of 190 patients with ischemic heart failure and confirmed LV thrombus were included; 86 (45.3%) demonstrated 6-month thrombus regression (Group 1) and 104 (54.7%) had no regression (Group 2).

### Baseline Comparisons by 6-month Thrombus Status

At baseline, the no-regression group showed a greater thrombus burden, higher vascular risk, and lower systolic function compared with the regression group: thrombus size (area method) was 3.0 (0.4–30.0) vs. 2.2 (0.1–10.6) (*p* = 0.045), the CHA₂DS₂-VA score was 3.5 ± 1.5 vs. 2.8 ± 1.3 (*p* = 0.001), and LVEF was 32.3 ± 7.2% vs. 35.0 ± 7.9% (*p* = 0.015). Other baseline characteristics were broadly similar between groups (Table 1).

**Table 1:** Baseline demographic, clinical, laboratory, and echocardiographic characteristics by 6-month LV thrombus response (Group 1: regression; Group 2: no regression).

Variables	Group 1	Group 2	*P* value
Age (years)	62.09 ± 12.75	66.81 ± 12.14	0.01
Sex (female) (n, %)	31 (36.0%)	18 (17.3%)	0.003
Hypertension (n, %)	47 (54.7%)	61 (58.7%)	0.579
Diabetes mellitus (n, %)	27 (31.4%)	41 (39.4%)	0.251
Vascular disease (n, %)	7 (8.1%)	5 (4.8%)	0.347
History of stroke (n, %)	12 (14.0%)	16 (15.4%)	0.782
Troponin elevation (n, %)	50 (58.1%)	59 (56.7%)	0.845
Rhythm (AF) (n, %)	14 (16.3%)	31 (29.8%)	0.029
Renal insufficiency (n, %)	7 (8.1%)	19 (18.3%)	0.043
History of bleeding (n, %)	3 (3.5%)	0 (0.0%)	0.055
Presence of pacemaker (n, %)	11 (12.8%)	7 (6.7%)	0.156
pre-antiplatelet use (n, %)	58 (67.4%)	74 (71.2%)	0.58
pre-anticoagulant use (n, %)	37 (43.0%)	33 (31.7%)	0.108
Creatinine (mg/dL)	1.01 (0.50–6.96)	1.10 (0.49–4.89)	0.138
eGFR (mL/min/1.73 m²)	72.09 ± 30.43	69.48 ± 26.71	0.534
Potassium (mmol/L)	4.60 (3.10–7.70)	4.50 (3.10–5.80)	0.197
Hemoglobin (g/dL)	13.19 ± 2.25	13.56 ± 2.20	0.258
Sodium (mmol/L)	138.00 (129.3–155)	139.00 (123.0–149.0)	0.191
Platelet count (×10³/µL)	262.34 ± 87.86	299.02 ± 95.96	0.007
Calcium (mg/dL)	9.09 ± 0.69	8.87 ± 0.70	0.065
Monocyte count (×10³/µL)	0.70 (0.09–1.60)	0.70 (0.10–2.70)	0.619
Lymphocyte count (×10³/µL)	2.00 (0.40–12.40)	1.97 (0.40–5.05)	0.813
Neutrophil count (×10³/µL)	5.40 (2.70–22.20)	5.75 (1.60–25.50)	0.838
White blood cell count (×10³/µL)	8.60 (4.40–25.00)	8.65 (2.90–28.00)	0.839
LDL (mg/dL)	91.00 (22.40–243.00)	88.20 (25.70–205.00)	0.366
HDL (mg/dL)	39.34 ± 10.57	38.20 ± 13.09	0.527
Triglycerides (mg/dL)	123.40 (41.00–364.00)	126.75 (56.00-674.60)	0.727
Troponin (hs-cTn)	0.24 (0.01–564.00)	0.20 (0.01–2627.00)	0.809
Heart rate (beats/min)	74.50 (50.00–132.00)	75.00(51.00–136.00)	0.344
Ejection fraction (%)	35.03 ± 7.85	32.32 ± 7.24	0.015
Left atrial diameter (mm)	36.33 ± 7.44	41.21 ± 6.39	< 0.001
LVEDD (mm)	52.31 ± 6.76	56.05 ± 6.86	< 0.001
LVESD (mm)	36.35 ± 9.47	40.88 ± 9.59	0.001
sPAP (mmHg)	29.50 (21.00–72.00)	35.00 (22.00–75.00)	< 0.001
Mitral E wave velocity (cm/s)	71.57 ± 24.74	70.60 ± 22.59	0.779
Mitral A-wave velocity (cm/s)	70.00 (-40.00–116.00)	75.00 (25.00–120.00)	0.604
Thrombus size (area methods, cm²)	2.16 (0.09–10.60)	2.97 (0.35–29.97)	0.045
CHA2DS2-VA score	2.80 ± 1.33	3.49 ± 1.49	< 0.001
1-year MACE (n, %)	11 (12.8%)	18 (17.3%)	0.389

Data are expressed as mean ± SD for normally distributed variables and median (min–max) for non-normally distributed variables. Between-group comparisons used independent-samples t test (normal), Mann–Whitney U test (non-normal), and Pearson’s χ² test for categorical variables (reported as n (%)). *p* < 0.05 was considered statistically significant

*AF*, atrial fibrillation; *eGFR*, estimated glomerular filtration rate; *LVEDD*, left ventricular end-diastolic diameter; *LVESD*, left ventricular end-systolic diameter; *MACE*, major adverse cardiovascular events; *PAP*, pulmonary artery pressure; *HDL/LDL*, high/low-density lipoprotein

### Model for Thrombus Non-regression at 6 months

In a multivariable logistic regression including thrombus size and CHA₂DS₂-VA, CHA₂DS₂-VA remained an independent predictor of non-regression (adjusted OR 1.35, 95% CI 1.09–1.69; *p* = 0.007), whereas thrombus size showed a positive, borderline association (adjusted OR 1.10, 95% CI 0.98–1.24; *p* = 0.103). The model’s apparent discrimination was AUC = 0.644.(Table 2).

**Table 2:** Univariable and multivariable logistic regression analyses for predictors of 1-year major adverse cardiovascular events (MACE)

Univariable Logistic Regression	OR	95% CI	*P* value
Thrombus size (area methods, cm²)	1.19	1.07–1.33	0.001
CHA2DS2-VA score	1.75	1.30–2.36	< 0.001
History of stroke	5.18	2.10-12.77	< 0.001
Sex (male)	3.36	1.48–7.62	0.004
Monocyte count	4.23	1.41–12.66	0.01
Renal insufficiency	3.03	1.17–7.83	0.022
Ejection fraction	0.94	0.89–0.99	0.03
eGFR	0.98	0.97-1.00	0.036
Multivariable Logistic Regression			
Thrombus size (area methods, cm²)	1.14	1.03–1.27	0.016
CHA2DS2-VA score	1.57	1.14–2.15	0.005

Sample size *n* = 190; number of events = 29; model discrimination AUC = 0.713

Odds ratios reflect the change per 1-unit increase in each continuous predictor. MACE indicates major adverse cardiovascular events; CI, confidence interval; OR, odds ratio

### One-year Outcomes

**Fig. 1:**
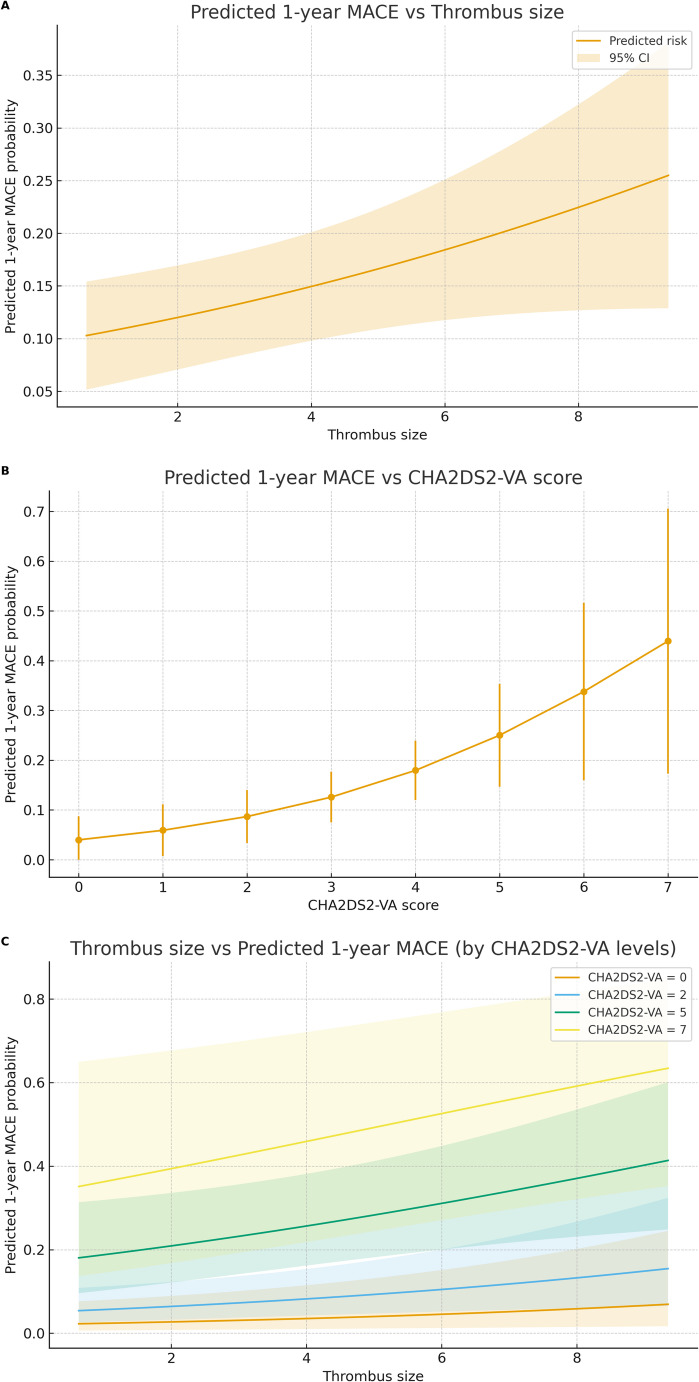
Panel **A**–**C**. Summary of prediction displays. (**A**) Predicted 1-year MACE vs. thrombus size (marginal). (**B**) Predicted 1-year MACE vs. CHA₂DS₂-VA score (marginal). (**C**) Thrombus size vs. predicted 1-year MACE stratified by CHA₂DS₂-VA level (conditional)

The incidence of 1-year MACE was 11/86 (12.8%) in Group 1 vs. 18/104 (17.3%) in Group 2 (*p* = 0.510), indicating no significant difference by thrombus regression status. In a separate multivariable model for 1-year MACE, both thrombus size (adjusted OR 1.14, 95% CI 1.03–1.27; *p* = 0.016) and CHA₂DS₂-VA (adjusted OR 1.57, 95% CI 1.14–2.15; *p* = 0.005) were associated with higher risk; model discrimination was AUC = 0.713 (Fig. 1).

### Machine Learning Outcomes

**Fig. 2:**
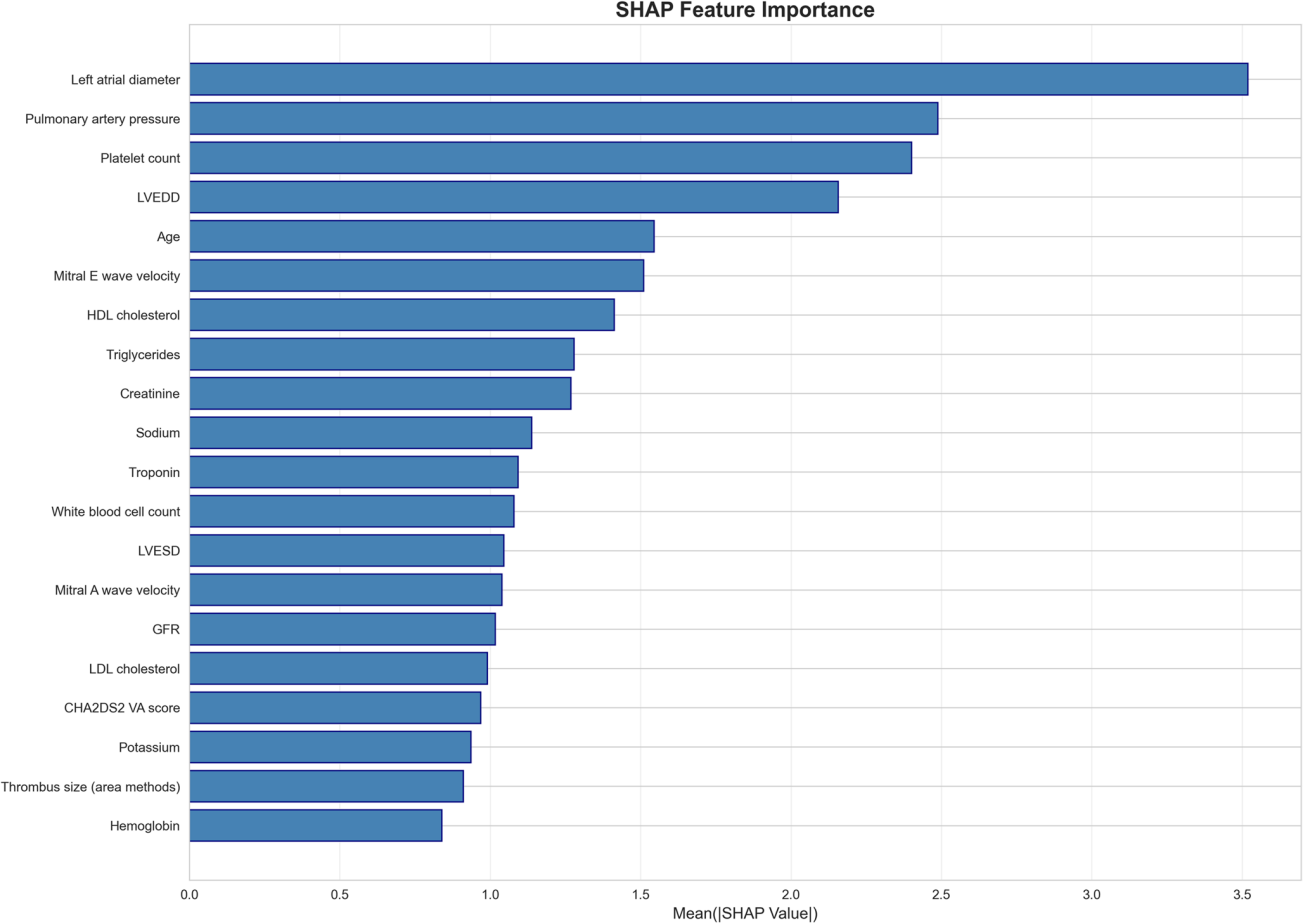
Global SHAP feature importance—Top-20 baseline predictors (model-agnostic display). Overall ranking of the top 20 baseline predictors by mean(|SHAP value|). The list again prioritizes left atrial diameter, pulmonary artery pressure, platelet count, and LVEDD, with metabolic/renal markers (e.g., triglycerides, creatinine, sodium) contributing smaller but consistent signals. Results are averaged on the evaluation split; details of imputation, scaling, encoding, and resampling are provided in Methods

**Fig. 3:**
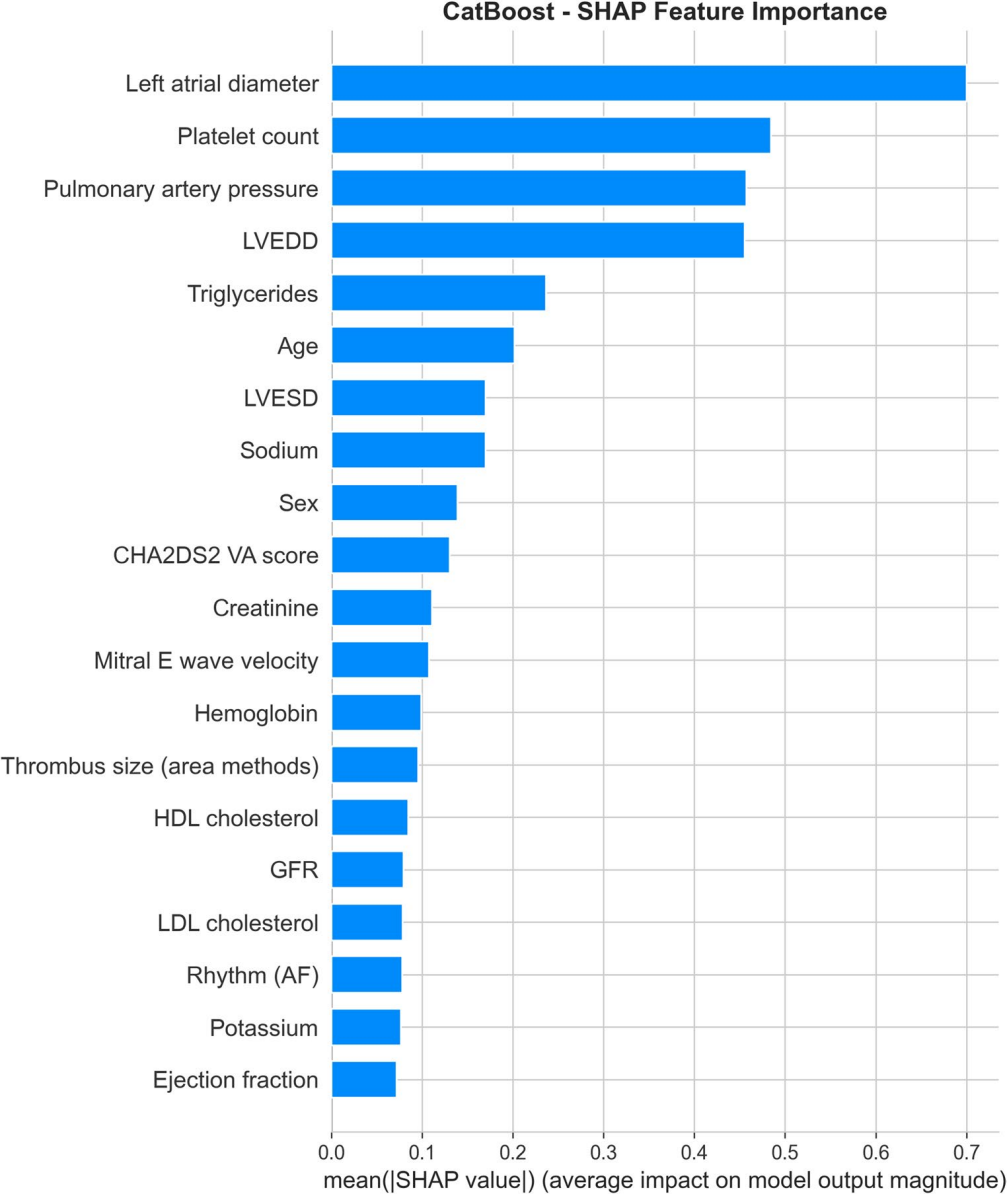
CatBoost—Global SHAP feature importance for 6-month LV thrombus non-regression. Global importance ranked by mean(|SHAP value|) on the hold-out data; higher bars indicate greater average contribution to the model’s predictions of non-regression (Group 2). Top predictors were left atrial diameter, platelet count, pulmonary artery pressure, and LVEDD, whereas CHA₂DS₂-VA score and thrombus size (area method) ranked lower—highlighting complementary hemodynamic/remodeling and hematologic signals beyond thrombus burden and clinical risk score. Pre-processing and cross-validation are described in Methods

**Fig. 4:**
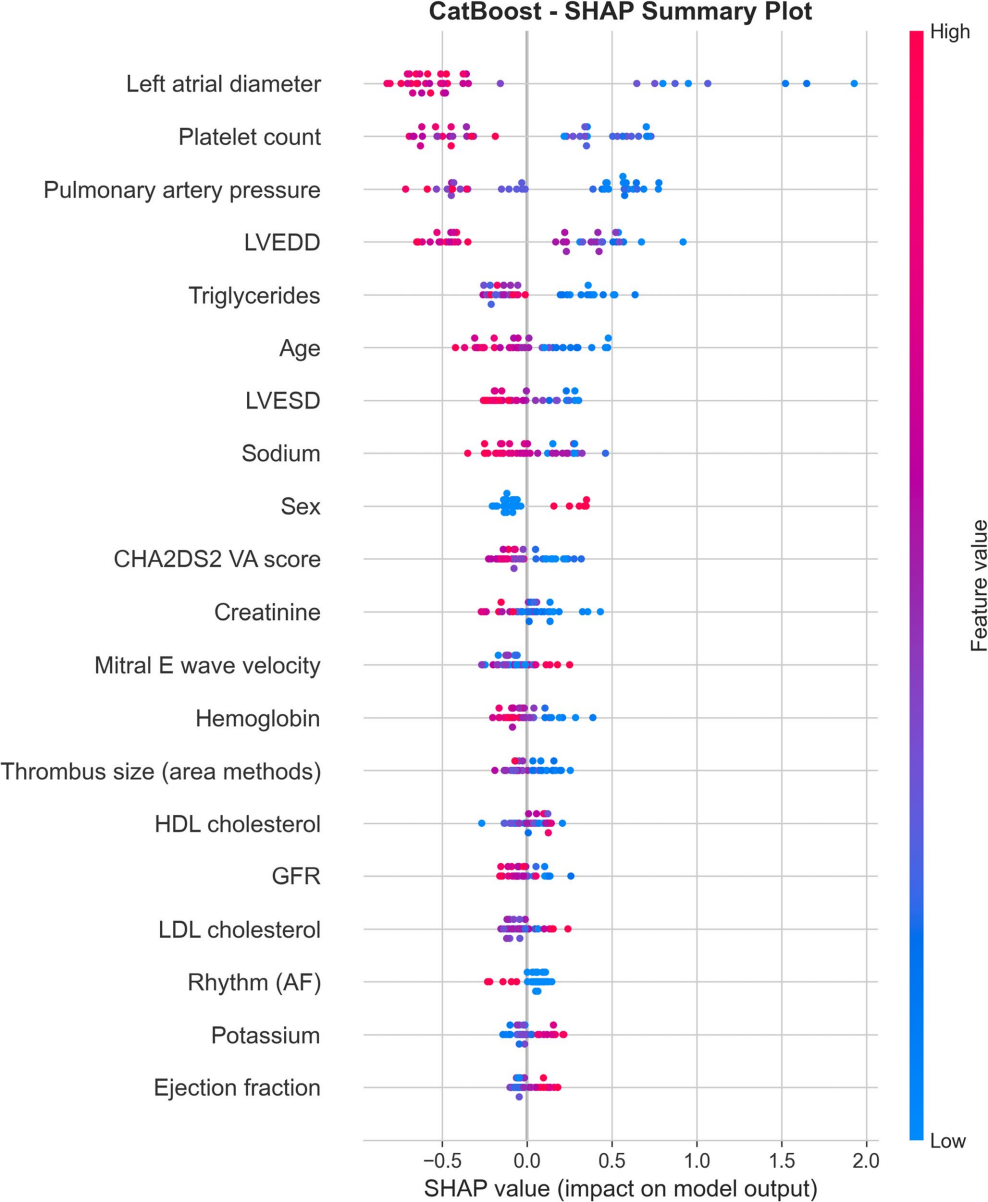
CatBoost—SHAP summary (beeswarm) plot for 6-month LV thrombus non-regression. Each dot represents one patient; the x-axis shows the SHAP value (impact on the log-odds of non-regression), with positive values shifting the prediction toward non-regression and negative values toward regression. Dot color encodes the feature value (red = high, blue = low). Higher left atrial diameter, pulmonary artery pressure, LVEDD, and platelet count cluster to the right (risk-increasing), whereas higher ejection fraction tends to shift predictions left (protective). This pattern supports a multidimensional risk phenotype (stasis/remodeling + hypercoagulability)

A total of three models were compared. CatBoost achieved the highest separation in internal validation (CV-AUC 0.762 ± 0.055); In the test set, accuracy 0.789, F1 0.733, precision 0.846 and recall 0.647 were achieved. (Table 3; Fig. 2) Random Forest showed a similar discriminating power (AUC ≈ 0.762), while XGBoost offered moderate performance with AUC ≈ 0.723. Confusion matrices showed that CatBoost kept false positives limited (high precision) while keeping false negatives moderate (recall); This suggests that it would be appropriate to set the threshold according to the error profile to be preferred in clinical use. Left atrial diameter, pulmonary artery pressure, platelet count, and LVEDD took the first places in the feature importance/SHAP analyses; these were followed by age, mitral E wave rate, HDL, triglycerides, creatinine and sodium. SHAP distributions showed that LA diameter and PAP elevation and LVEDD increase provided directional contributions that increased the likelihood of non-regression, while platelet count provided a complementary signal reflecting the hypercoagulability axis. (Figures 3 and 4) In contrast, CHA₂DS₂-VA and thrombus size ranked lower in ML models; this is consistent with hemodynamics/stasis and remodeling markers (LA diameter, PAP, LVEDD) better capturing nonlinear thresholds/interactions within the data and redistributing the common variance to these axes. Overall, ML provided a modest increase in discrimination compared to partial logistics and showed that risk can be better captured through a multidimensional phenotype in which stasis-remodeling-coagulability components are evaluated together, not just “thrombus + score”.

**Table 3:** Comparative performance metrics of machine-learning models for predicting left ventricular thrombus resolution

	Accuracy	Precision	Recall	F1-Score	AUC
Random Forest	0.7632	0.75	0.7059	0.7273	0.7619
XGBoost	0.7105	0.6667	0.7059	0.6857	0.7227
CatBoost	0.7895	0.8462	0.6471	0.7333	0.7535

*AUC*, area under the receiver operating characteristic curve

F1-score represents the harmonic mean of precision and recall

## Discussion

In this study, machine learning offered a unique perspective that complements the classical statistical approach to predicting the risk of non-regression at 6 months. Our partial logistic model (thrombus size + CHA₂DS₂-VA) provided reasonable separating power (AUC ≈ 0.64), while tree-based ML methods (Random Forest, XGBoost, CatBoost) achieved a moderate additional segregation power by capturing nonlinear threshold/interaction patterns. Importantly, the variable significance profile of ML provided a different clinical outlook than classical analyses: left atrial diameter, pulmonary artery pressure, platelet count, and LVEDD were at the forefront, while CHA₂DS₂-VA and thrombus size were relatively lower. The lack of a clear 1-year MACE difference based on the regression analysis results between the two groups advocates multiparameter risk stratification rather than relying solely on imaging response.

The prominence of left atrial diameter, sPAP, platelet count, and LVEDD in machine learning models was in line with an expected pattern in terms of the pathophysiology of the LV thrombus. Pro-thrombotic changes in stasis and blood components, which are the two legs of the Virchow triad, are indirectly captured in these four indicators. The enlargement of the left atrium reflects chronic elevated filling pressure and atrial myopathy; this is associated with slowing of atrial flow/stasis and has been shown in many studies to increase the risk of thromboembolism (regardless of the presence of AF). The association of left atrial enlargement and/or dysfunction with stroke risk; It has been reported recurrentantly in older populations, AF cohorts and the general population. The LA diameter can therefore act as an indirect “high filling pressure/stasis” biomarker in advanced geometry, where intraventricular stasis is also exacerbated [10–13]. 

Increased sPAP is a reflection of post-capillary pulmonary hypertension, most often due to left heart disease, and indicates the retransmission of chronically elevated LA pressure to the pulmonary circulation. This hemodynamic background is characterized by higher left ventricular filling pressure, more pronounced structural remodeling, and lower forward flow; As a result, the tendency of blood to wait in the ventricle (stasis) and increased thrombus organization/persistence are reasonable. The literature indicates that PH due to left heart disease is common, prognostically unfavorable, and closely associated with elevated LA pressure; suggesting that PAP may be a mediator for “disease severity and stasis” within the model [14, 15]. 

The prominence of platelet count, on the other hand, supports the hypercoagulability arm. Increased platelet activity/reactivity in the context of acute MI and ischemic heart failure has been associated with the development of LVT and/or delayed thrombus resolution; these studies strengthen the role of platelet biology in the pathogenesis of LVT and suggest that numerical load and reactivity indicators may contribute to risk stratification [16–18]. 

On the other hand, LVEDD elevation reflects adverse ventricular remodeling, large cavity, apical akinesia/aneurysm, and low EF spectrum; This combination of anatomy and function enhances intraventricular stasis, creating a favorable environment for LVT formation and persistence. In CMR and echocardiography-based series, LVT was found to be more frequently detected, especially with anterior wall MI, low EF, and adverse remodeling (larger LV sizes); It has been reported that the volume of LVEDD, LVESD and LA is larger in persistent LVT [19, 20]. Within this totality, it seems biologically consistent that our models attribute high importance to these four variables; However, it should be noted that variables may be proxies for disease severity and hemodynamic phenotype rather than causal effect.

In our study, we found that the strongest signal for 1-year MACE came from baseline clinical and thrombotic load: thrombus size and CHA₂DS₂-VA score were independently correlated with MACE in the multivariate model (AUC ≈ 0.71). This finding is consistent with the literature that the presence of LV thrombus and thrombus burden increase the risk of embolic events and adverse cardiovascular outcomes. As a matter of fact, guideline proceedings and cohort studies show that LV thrombus carries a high risk of embolism and MACE when left untreated/not properly managed; It was also reported that providing regression with ≥ 3 months of anticoagulation and imaging may be associated with a reduction in total risk [21–23]. Although there was no significant difference between the “regression vs. persistence” groups in terms of 1-year MACE at 6 months, the limited number of events and the fact that anticoagulation compensates for the risk of embolism in the early period may have diluted this difference; on the other hand, the initial phenotype (thrombus size + vascular risk burden) captured the MACE signal more stably.

The association of CHA₂DS₂-VASc with MACE is consistent with the fact that the components in the score (advanced age, hypertension, diabetes, history of stroke/ischemic disease, etc.) collectively reflect a tendency for atherothrombotic events, supported by studies reporting that this score predicts adverse events in non-AF conditions (especially STEMI/ACS) [24]. In our data, the association of variables such as stroke history, renal failure/low eGFR and lower EF with MACE at the univariate level is in line with the large body of evidence showing that cardiovascular risk is strengthened by neuro-vascular and renal axes [25, 26]. In addition, the link between inflammatory cell indicators and event risk suggests the role of the thromboinflammation axis; This axis gave a weak but consistent signal in our model as well. From a clinical point of view, the interpretation is that the combination of simple scores (CHA₂DS₂-VA) and thrombus characteristics at baseline may provide an early and feasible risk stratification for 1-year MACE. LVT-focused tools (e.g., LEFT-type scores) may offer incremental value; however, required variables were not consistently available in our dataset. Comparative evaluation of general vascular-risk scores versus LVT-specific scores should be pursued in externally validated cohorts.

From a clinical point of view, these frameworks offer us a strategy based on the initial risk profile (thrombus characteristics + simple clinical scores + hemodynamics/shaping markers) rather than a response-oriented follow-up immediately after starting treatment. A simple score that is easy to apply at the bedside (e.g., thrombus size and a few clinical variables) can be maintained for baseline screening; When parameters such as LA diameter, PAP, LVEDD, and platelet count are high, ML-based stimulation may come into play for close imaging monitoring and more careful individualization of anticoagulation time/intensity.

## Limitations

Because this study was unicentric and retrospective, the possibility of selection bias and residual confounding effects cannot be completely excluded; causality cannot be inferred due to observational design. The limited sample size and especially the number of events may have reduced the statistical power in some comparisons and made it difficult to detect effects, especially in the subgroup/borderline. Because the type, dose, and duration of anticoagulation, as well as concomitant antithrombotic therapies, are variable with respect to clinical practice, treatment heterogeneity may have been reflected in model predictions. Since echocardiography is the primary method of detection and monitoring of LV thrombus (contrast use and frequency of measurements may vary according to clinical need), there is a risk of measurement bias and misclassification due to interim observation intervals (especially interval-censoring for the actual timing of regression); Since systematic CMR evaluation is not performed in all patients, it is possible that small/laminated thrombi may be missed. Thrombus size was assessed by area/planimetry method and does not directly capture three-dimensional volume or texture features (degree of organization, mobility); inter/intra-observer variability has not been formally tested. Although prediction models are partially designed and internal verification is performed, external verification and calibration are lacking; Since machine learning models are trained on a limited sample, they carry the risk of overfitting, and “feature importance” outputs are not causal. Finally, because the cohort focused on the ischemic heart failure phenotype, the findings may not be directly transferred to non-ischemic cardiomyopathies or different care settings for overall generalizability. BNP/NT-proBNP was not available for all patients in a standardized manner and was therefore not included; future prospective work should incorporate natriuretic peptides for model refinement and validation.

## Conclusion

In patients with LV thrombus in ischemic heart failure, both the risk of thrombus non-regression at 6 months and the possibility of 1-year MACE can be predicted with simple clinical and imaging markers at baseline. In particular, the CHA₂DS₂-VA score stood out as an independent marker for non-regression, while thrombus size showed borderline significance in terms of non-regression; on the other hand, both thrombus size and CHA₂DS₂-VA were found to be significantly associated for 1-year MACE. Machine learning analyses have shown that risk can be better captured through a multidimensional phenotype, emphasizing the importance of parameters that reflect stasis, remodeling, and hypercoagulability, such as left atrial diameter, pulmonary artery pressure, platelet count, and LVEDD, to complement classical models. Clinically, these findings support an approach in which thrombus size, simple clinical scores, hemodynamic/remodeling and hematological markers are used together, and close imaging monitoring and anticoagulation time/intensity are individualized.

## Data Availability

The datasets generated and/or analyzed during the current study are not publicly available due to patient confidentiality and institutional restrictions, but are available from the corresponding author on reasonable request.

## References

[1] Levine GN, McEvoy JW, Fang JC, et al. Management of Patients at Risk for and With Left Ventricular Thrombus: A Scientific Statement From the American Heart Association. Circulation. 2022;146(15):e205–23. 10.1161/CIR.0000000000001092. 36106537 10.1161/CIR.0000000000001092

[2] Phuah Y, Tan YX, Zaghloul S, et al. A systematic review and meta-analysis of transthoracic echocardiogram vs. cardiac magnetic resonance imaging for the detection of left ventricular thrombus. Eur Heart J Imaging Methods Pract. 2023;1(2):qyad041. 10.1093/ehjimp/qyad041. Published 2023 Dec 7. 39045058 10.1093/ehjimp/qyad041 PMC11240154

[3] Haller PM, Kazem N, Agewall S, et al. Oral anticoagulation in patients with left ventricular thrombus: a systematic review and meta-analysis. Eur Heart J Cardiovasc Pharmacother. 2024;10(5):444–53. 10.1093/ehjcvp/pvae042. 38845369 10.1093/ehjcvp/pvae042

[4] Goh FQ, Sia CH, Chan MY, Yeo LL, Tan BY. What’s the optimal duration of anticoagulation in patients with left ventricular thrombus? Expert Rev Cardiovasc Ther. 2023;21(12):947–61. 10.1080/14779072.2023.2270906. 37830297 10.1080/14779072.2023.2270906

[5] Dai Y, Ouyang C, Luo G, et al. Risk factors for high CAD-RADS scoring in CAD patients revealed by machine learning methods: a retrospective study. PeerJ. 2023;11:e15797. 10.7717/peerj.15797. Published 2023 Aug 3. 37551346 10.7717/peerj.15797 PMC10404399

[6] Sritharan HP, Nguyen H, Ciofani J, Bhindi R, Allahwala UK. Machine-learning based risk prediction of in-hospital outcomes following STEMI: the STEMI-ML score. Front Cardiovasc Med. 2024;11:1454321. 10.3389/fcvm.2024.1454321. Published 2024 Oct 10. 39450234 10.3389/fcvm.2024.1454321 PMC11499125

[7] Leha A, Huber C, Friede T, et al. Development and validation of explainable machine learning models for risk of mortality in transcatheter aortic valve implantation: TAVI risk machine scores. Eur Heart J Digit Health. 2023;4(3):225–35. 10.1093/ehjdh/ztad021. Published 2023 Mar 17. 37265865 10.1093/ehjdh/ztad021 PMC10232286

[8] Liu Y, Chen Y, Olier I, et al. Residual risk prediction in anticoagulated patients with atrial fibrillation using machine learning: A report from the GLORIA-AF registry phase II/III. Eur J Clin Invest. 2025;55(3):e14371. 10.1111/eci.14371. 39660499 10.1111/eci.14371 PMC11810544

[9] Zhang Q, Zheng H, Zhang Z, Xu Y, Zhang W. Advancing clinical management of left ventricular thrombosis: prevention, detection and treatment modalities in the modern era. Heart. 2025;111(14):662–70. 10.1136/heartjnl-2024-324605. Published 2025 Jun 26. 39938942 10.1136/heartjnl-2024-324605 PMC12229065

[10] Parajuli P, Alahmadi MH, Ahmed AA. Left Atrial Enlargement. [Updated 2025 Jan 22]. In: StatPearls [Internet]. Treasure Island (FL): StatPearls Publishing; 2025 Jan-. Available from: https://www.ncbi.nlm.nih.gov/books/NBK553096/31971736

[11] Xu Y, Zhao L, Zhang L, Han Y, Wang P, Yu S. Left Atrial Enlargement and the Risk of Stroke: A Meta-Analysis of Prospective Cohort Studies. Front Neurol. 2020;11(26). 10.3389/fneur.2020.00026. Published 2020 Feb 14. 10.3389/fneur.2020.00026 PMC7033471 32117002

[12] Mannina C, Ito K, Jin Z, et al. Association of Left Atrial Strain With Ischemic Stroke Risk in Older Adults. JAMA Cardiol. 2023;8(4):317–25. 10.1001/jamacardio.2022.5449. 36753086 10.1001/jamacardio.2022.5449 PMC9909576

[13] Lin AY, Dinatolo E, Metra M, et al. Thromboembolism in Heart Failure Patients in Sinus Rhythm: Epidemiology, Pathophysiology, Clinical Trials, and Future Direction. JACC Heart Fail. 2021;9(4):243–53. 10.1016/j.jchf.2021.01.009. 33714744 10.1016/j.jchf.2021.01.009

[14] Vachiéry JL, Tedford RJ, Rosenkranz S, et al. Pulmonary hypertension due to left heart disease. Eur Respir J. 2019;53(1):1801897. 10.1183/13993003.01897-2018. Published 2019 Jan 24. 30545974 10.1183/13993003.01897-2018 PMC6351334

[15] Riley JM, Fradin JJ, Russ DH, Warner ED, Brailovsky Y, Rajapreyar I. Post-Capillary Pulmonary Hypertension: Clinical Review. J Clin Med. 2024;13(2):625. 10.3390/jcm13020625. Published 2024 Jan 22. 38276131 10.3390/jcm13020625 PMC10816629

[16] Acar Z, Ziyrek M, Korkmaz L, Kiris A, Sahin S, Celik S. Mean platelet volume at admission is a determinant of left ventricular thrombus formation after primary percutaneous coronary intervention for first anterior wall myocardial infarction. Acta Cardiol. 2014;69(6):603–9. 10.2143/AC.69.6.1000002. 25643430 10.2143/AC.69.6.1000002

[17] Zhang Q, Si D, Zhang Z, et al. Value of the platelet-to-lymphocyte ratio in the prediction of left ventricular thrombus in anterior ST-elevation myocardial infarction with left ventricular dysfunction. BMC Cardiovasc Disord. 2020;20(1):428. 10.1186/s12872-020-01712-w. Published 2020 Sep 29. 32993501 10.1186/s12872-020-01712-w PMC7526106

[18] Sia CH, Leow AS, Tan BY, et al. The neutrophil-lymphocyte ratio and platelet-lymphocyte ratio predict left ventricular thrombus resolution in acute myocardial infarction without percutaneous coronary intervention. Thromb Res. 2020;194:16–20. 10.1016/j.thromres.2020.06.003. 32559523 10.1016/j.thromres.2020.06.003

[19] Phan J, Nguyen T, French J, et al. Incidence and predictors of left ventricular thrombus formation following acute ST-segment elevation myocardial infarction: A serial cardiac MRI study. Int J Cardiol Heart Vasc. 2019;24:100395. 10.1016/j.ijcha.2019.100395. Published 2019 Jul 4. 31321288 10.1016/j.ijcha.2019.100395 PMC6612928

[20] Salah Shabib Ahmed H, Ede H, Sobhy Hassan Ghonim Mahfouz A, et al. Surrogates of the Left Ventricular Thrombus Resolution: A Retrospective Data Review. Turk Kardiyol Dern Ars. 2022;50(3):168–74. 10.5543/tkda.2022.21068. 35450840 10.5543/tkda.2022.21068

[21] Cregler LL. Antithrombotic therapy in left ventricular thrombosis and systemic embolism. Am Heart J. 1992;123(4 Pt 2):1110–4. 10.1016/0002-8703(92)91069-d. 1553880 10.1016/0002-8703(92)91069-d

[22] Lattuca B, Bouziri N, Kerneis M, et al. Antithrombotic Therapy for Patients With Left Ventricular Mural Thrombus. J Am Coll Cardiol. 2020;75(14):1676–85. 10.1016/j.jacc.2020.01.057. 32273033 10.1016/j.jacc.2020.01.057

[23] Velangi PS, Choo C, Chen KA, et al. Long-Term Embolic Outcomes After Detection of Left Ventricular Thrombus by Late Gadolinium Enhancement Cardiovascular Magnetic Resonance Imaging: A Matched Cohort Study. Circ Cardiovasc Imaging. 2019;12(11):e009723. 10.1161/CIRCIMAGING.119.009723. 31707810 10.1161/CIRCIMAGING.119.009723 PMC6941143

[24] Bozbay M, Uyarel H, Cicek G, et al. CHA_2_DS_2_-VASc Score Predicts In-Hospital and Long-Term Clinical Outcomes in Patients With ST-Segment Elevation Myocardial Infarction Who Were Undergoing Primary Percutaneous Coronary Intervention. Clin Appl Thromb Hemost. 2017;23(2):132–8. 10.1177/1076029616646874. 27170782 10.1177/1076029616646874

[25] Ashoori A, Pourhosseini H, Ghodsi S, et al. CHA2DS2-VASc Score as an Independent Predictor of Suboptimal Reperfusion and Short-Term Mortality after Primary PCI in Patients with Acute ST Segment Elevation Myocardial Infarction. Med (Kaunas). 2019;55(2):35. 10.3390/medicina55020035. Published 2019 Feb 1. 10.3390/medicina55020035 PMC6409514 30717292

[26] Jankowski J, Floege J, Fliser D, Böhm M, Marx N. Cardiovascular Disease in Chronic Kidney Disease: Pathophysiological Insights and Therapeutic Options. Circulation. 2021;143(11):1157–72. 10.1161/CIRCULATIONAHA.120.050686. 33720773 10.1161/CIRCULATIONAHA.120.050686 PMC7969169

